# Fruit and vegetable consumption, dietary and plasma carotenoids and risks of recurrence and progression in patients with non-muscle-invasive bladder cancer

**DOI:** 10.1007/s00394-026-04079-4

**Published:** 2026-08-21

**Authors:** Alina Vrieling, Joann Kiebach, Michiel Balvers, Katja K. Aben, Antoine G. van der Heijden, Ellen Kampman, Lambertus A. Kiemeney

**Affiliations:** 1 IQ Health Science Department (HP 160), Radboud University Medical Center, Kapittelweg 54, 6525 EP Nijmegen, The Netherlands; 2 Department of Primary and Community Care, Radboud University Medical Center, Nijmegen, The Netherlands; 3 Division of Human Nutrition and Health, Wageningen University & Research, Wageningen, The Netherlands; 4 Department of Research and Development, Netherlands Comprehensive Cancer Organisation, Utrecht, The Netherlands; 5 Department of Urology, Radboud University Medical Center, Nijmegen, The Netherlands

**Keywords:** Fruit and vegetables, Carotenoids, Biomarkers, Patients with non-muscle invasive bladder cancer, Recurrence, Progression

## Abstract

**Purpose:**

Fruit and vegetable consumption may decrease bladder cancer risk, but research on its relation to outcomes of non-muscle-invasive bladder cancer (NMIBC) is scarce. We investigated the association of (changes in) pre- and postdiagnosis fruit and vegetable consumption, as well as postdiagnosis dietary and plasma carotenoids, with risks of recurrence and progression in patients with NMIBC.

**Methods:**

Data from patients with newly diagnosed NMIBC between 2014 and 2021 from the prospective multi-centre cohort UroLife was used. Patients reported prediagnosis diet (*n* = 1396), and diet at 3 and 15 months postdiagnosis (*n* = 1270). Plasma carotenoid concentrations were measured at 3 and 15 months postdiagnosis (*n* = 910). Multivariable proportional hazards regression analyses were performed to assess associations with risks of first recurrence, multiple recurrences, and progression.

**Results:**

Median follow-up time was 4.7 years, 466 patients had at least one recurrence, 139 had multiple recurrences, and 153 showed progression. Total fruit and vegetable consumption at 3 months postdiagnosis as well as pre-to-postdiagnosis increases in consumption were inversely associated with first recurrence risk (per 100 g/day: hazard ratio [HR] = 0.91, 95% confidence interval ([95%CI] 0.85–0.99 and HR = 0.90, 95%CI 0.81–0.99, respectively). Results were attenuated at 15 months after diagnosis. Total and individual dietary and plasma carotenoids were not associated with first recurrence risk. No associations with multiple recurrences and progression, and for prediagnosis fruit and vegetable consumption were found.

**Conclusion:**

Higher postdiagnosis fruit and vegetable consumption may have a beneficial role in NMIBC recurrence but not progression risk while no associations for dietary and plasma carotenoids were found.

**Supplementary Information:**

The online version contains supplementary material available at https://doi.org/10.1007/s00394-026-04079-4.

## Introduction

Bladder cancer (BC) is the ninth most common cancer worldwide [1], and approximately 75% of patients are diagnosed with non-muscle-invasive bladder cancer (NMIBC) [2]. These patients have a good overall survival but their risk of tumour recurrence and disease progression is high [3]. Therefore, it is important to identify modifiable factors that may decrease recurrence and progression risk.

Most studies focusing on BC evaluated the role of diet and other lifestyle factors in BC prevention. In 2018, the World Cancer Research Fund/American Institute for Cancer Research (WCRF/AICR) report concluded that there is limited suggestive evidence that fruit and vegetable consumption is associated with decreased BC risk [4]. More recently, a meta-analysis including 15 prospective cohort studies also revealed inverse associations of fruit and vegetable consumption with BC risk, although these did not reach statistical significance [5].

Fruit and vegetables are hypothesized to help prevent cancer due to their high content of vitamins and phytochemicals, including carotenoids [6]. Carotenoids have antioxidant and immunomodulatory properties and have been related to decreased cancer risk [7]. They are extensively metabolized and are, as well as their metabolites, partly excreted in the urine [8]. Carotenoids have also been associated with decreased BC risk [9].

So far, only three studies investigated the association of consumption of fruit and vegetables in relation to risk of NMIBC recurrence and/or progression. One relatively small study investigated pre- (*n* = 728) and postdiagnosis (*n* = 389) fruit and vegetable intake [10], one relatively small study (*n* = 595) examined a prediagnosis fruit and vegetables dietary pattern [11], and one study (*n* = 1143) only focused on cruciferous vegetables intake and related biomarkers shortly after diagnosis [12]. Results were inconsistent, and studies on pre- to postdiagnosis changes in fruit and vegetable consumption and postdiagnosis dietary and circulating carotenoids are lacking. Here, we investigated the association of (changes in) pre- and postdiagnosis fruit and vegetable consumption as well as postdiagnosis dietary and plasma carotenoids with risks of recurrence and progression in the largest prospective cohort study on diet and clinical outcomes among patients with NMIBC to date.

## Methods

### Study design

This study was conducted using data from the multicenter prospective cohort study UroLife, which has been described in detail elsewhere [13]. In short, the UroLife study included patients with newly diagnosed NMIBC from 22 hospitals in The Netherlands between May 2014 and April 2021. Eligible patients were aged between 18 and 80 years, Dutch-speaking, and underwent a transurethral resection of a bladder tumour (TURBT) with histologically confirmed Ta, T1, or Tis NMIBC (only stage T1 from May 2017 onwards). Participants were asked to complete web- or paper-based questionnaires at 6 weeks, 3, 15 and 51 months after diagnosis. The assessment at 51 months was excluded from this analysis, as most recurrences and progression occurred earlier. Blood samples were collected at 3 and 15 months after diagnosis. Detailed information on exclusion criteria is shown in Supplementary Figs. 1 and 2. All participants provided written informed consent. The Committee for Human Research region Arnhem-Nijmegen provided ethical approval (CMO 2013 − 494). The study was performed in accordance with the ethical standards as laid down in the 1964 Declaration of Helsinki.

### Fruit and vegetable intake assessment

Dietary intake was assessed using a validated 163-item food frequency questionnaire (FFQ) [14–16], with acceptable ability to rank subjects according to their fruit and vegetable intake [16]. Participants reported prediagnosis dietary intake at 6 weeks after diagnosis, and postdiagnosis dietary intake at 3 months after diagnosis (to capture short-term changes after initial treatment started) and 15 months after diagnosis (to capture more durable changes after diagnosis). The reference period was the previous year (prediagnosis; fruit and vegetable intake was asked for summer and winter, separately) or month (postdiagnosis). Dietary intake was determined by multiplying the frequency of the food item consumption by the standard serving size. The standard serving size of fruit and vegetables was defined as a serving spoon of 50 gram. Total fruit and vegetable intake was analysed in grams/day and was computed as the sum of all the fruit and vegetables items. Fruits included the items apple, banana, citrus fruit, strawberry, kiwi, and an item named other fresh fruits. Vegetables included the items cauliflower and broccoli, cabbage, spinach, onion, beans, carrot, lettuce, tomato, and an item named other cooked, stir-fried or raw vegetables. Nutrient intake, including carotenoids, was calculated using the Dutch Food Composition Database (in Dutch: NEVO-table) from 2011 [17].

### Plasma carotenoids assessment

Non-fasting heparin plasma samples were wrapped in aluminium foil immediately after blood collection to prevent degradation of carotenoids by light. Plasma samples were processed and stored at − 80 °C in the participating hospitals until transportation on dry ice to the Radboud Biobank. Here, the samples were stored at − 80 °C until transportation on dry ice to Wageningen University and Research for analysis of carotenoids. Carotenoids were only measured for participants included between April 2014 and April 2017. They were analysed in January 2019 (16 batches) and July 2019 (28 batches). For each participant, the plasma samples at 3 months and 15 months were analysed on the same plate in the same batch.

Carotenoid measurements were performed with High Performance Liquid Chromatography (Thermo Scientific Accela LC system; Thermo Fisher Scientific), and analyzed using Chromquest 5.0, V.3.2.1. software (Thermo Fisher Scientific). In total, 250 µL plasma, 250 µL NaCl (0.9 w/v% in water), and 500 µL ethanol (with added retinyl acetate as internal standard) were mixed and extracted twice with 1500 µL hexane. The hexane layers were pooled and evaporated to dryness in a vacuum concentrator at 35 °C (RVC 2.25 CD plus; MartinChrist). The residue was dissolved in a 150 µL mixture of methanol and butanol (60:40 vol: vol %); 25 µL was injected per HPLC analysis. Sample preparations were done under subdued yellow light. The carotenoids α- and β-carotene, β-cryptoxanthin, lycopene, lutein, and zeaxanthin were separated on a C18 reverse phase column (Vydac 201TP52, Hichrom, Lutterworth, UK) with a quard column (Vydac 201GD52) by using gradient elution, and monitored at 450 nm on a photodiode array detector.

For quality control, 2 control samples with concentrations similar to the mean concentrations in participants were added to each of the 30 batches of samples to assess intra- and inter-batch reproducibility. Intra-batch coefficients of variation (CVs) were 5.0% for α-carotene, 4.8% for β-carotene, 5.3% for lycopene, 4.5% for lutein, 4.3% for β-cryptoxanthin, and 6.2% for zeaxanthin. Inter-batch CVs were 8.0% for α-carotene, 8.5% for β-carotene, 15.9% for lycopene, 10.1% for lutein, 9.2% for β-cryptoxanthin, and 19.3% for zeaxanthin.

### Outcome assessment

The primary outcome was first recurrence, and multiple recurrences and progression were secondary outcomes. Outcome information was retrieved from medical records by trained data managers from the Netherlands Cancer Registry (NCR) between February–March 2021 and between April–July 2022. A recurrence was defined as the first new bladder tumour after being tumour-free. Patients with Ta and T1 tumours were considered tumour-free from the date of radical (primary or re-) TURBT, while patients with carcinoma in situ (CIS) or irradical primary or re-TURBT were considered tumour-free from the date of the first tumour-negative cystoscopy after TURBT. Progression was specified as an increase in grade or stage, or development of metastasis, as described in detail elsewhere [18]. Follow-up time started at the day of 6 weeks (prediagnosis) or 3 months (postdiagnosis) questionnaire completion / blood collection or, for those initially not tumour-free, at the first day of being-tumour free (for recurrence only). As the 15-month assessment likely reflects habitual fruit and vegetable consumption and dietary and plasma carotenoids after diagnosis, we decided to start the follow-up of the 15-month analyses at the same date as the 3-month analyses. Patients were censored at last follow-up visit, radical cystectomy in absence of recurrence or progression, diagnosis of cancer with metastasis, or progression to muscle-invasive bladder cancer (for multiple recurrences only), whichever came first [19].

### Covariate assessment

Information on age and sex was collected at 6 weeks after diagnosis. Clinical data was retrieved by the NCR and included tumour characteristics and treatment information. Participants were classified into a low-, intermediate-, or high-risk prognostic risk group based on primary tumour stage, grade, concomitant CIS, and focality, but not tumour size (mostly not registered in patient files), as defined by the 2019 guidelines of the European Association of Urology (EAU) [20]. Data on other characteristics, such as smoking status, BMI, moderate-to-vigorous physical activity, comorbidities, and energy intake were collected by questionnaires at all time points as previously described [13].

### Statistical analysis

Descriptive characteristics were calculated for the total study population and stratified by quartiles of fruit and vegetable consumption and tertiles of plasma carotenoid concentrations at 3 months. To assess the stability of fruit and vegetable consumption, dietary carotenoids, and plasma carotenoids at 15 compared to 3 months after diagnosis, intraclass correlation coefficients (ICCs) were calculated. Pearson correlation coefficients were calculated to assess the correlation of plasma carotenoids with fruit and vegetable consumption and dietary carotenoids.

Cox proportional hazards regression models were used to calculate hazard ratios (HRs) and 95% confidence intervals (95%CI) for the associations of (changes in) pre- and postdiagnosis fruit and vegetable consumption and postdiagnosis dietary and plasma carotenoids with risks of first recurrence and progression. The primary exposures of interest were postdiagnosis fruit and vegetable consumption and carotenoids, the secondary exposures of interest prediagnosis and changes in fruit and vegetable consumption. The change in fruit and vegetable consumption was calculated by subtracting the prediagnosis consumption from the postdiagnosis consumption. To investigate these associations for multiple recurrence risk, gap time-unrestricted (GT-UR) Cox models with random frailty terms were used, as previously described in detail [18]. HRs for fruit and vegetables were calculated per 100 g/day increase in intake. For dietary and plasma carotenoids, we performed a log-2 transformation to improve normality. A single step on the log-2 transformed scale represents a doubling of the dietary intake or plasma concentration of the carotenoids. Continuous associations were tested for non-linearity using likelihood ratio chi-square tests comparing multivariable models with and without restricted cubic splines (with knots placed at the 10th, 50th, and 90th percentiles, at the 5th, 35th, 65th, and 95th percentiles, or at the 5th, 27.5th, 50th, 72.5th and 95th percentiles, depending on the best model fit based on the Akaike Information Criterion [AIC]) [21].

A sufficient adjustment set (i.e. covariates) was selected from a directed acyclic graph (DAG) (Supplementary Fig. 3), which was based on prior knowledge [9, 22–26] and expert opinion. To estimate the causal association of fruit and vegetable consumption and dietary and plasma carotenoids with NMIBC recurrence and progression risk, the sufficient adjustment set included age at diagnosis, sex, smoking status (never, former, current), BMI (continuous), moderate-to-vigorous physical activity (continuous), energy intake (continuous). Missing data on fruit and vegetable consumption at 15 months (*n* = 199), missing plasma carotenoid concentrations at 3 (*n* = 18) or 15 months (*n* = 101), and missing covariates were imputed using multiple imputation by chained equations (MICE) with 20 iterations, including Nelson-Aalen estimators and survival outcomes. Complete case analyses were also performed and showed similar results (data not shown).

Stratified analyses for our primary exposure of interest were performed to explore potential effect modification of the association of postdiagnosis fruit and vegetable consumption and dietary and plasma carotenoids with risk of first recurrence, multiple recurrences and progression by sex, smoking status, tumour stage, EAU risk score, and Bacillus Calmette-Guérin (BCG) treatment.

All analyses were performed in R for Windows version 4.4.1 (R Foundation for Statistical Computing, Vienna, Austria), using the packages ‘MICE’, ‘rms’, ‘survival’, and ‘coxme’. A *P* ≤ 0.05 was considered statistically significant.

## Results

A total of 1396 and 1270 participants were included in the pre- and postdiagnosis analyses of fruit and vegetable consumption and recurrence risk, respectively (Supplementary Fig. 1), and a total of 910 participants in the postdiagnosis analyses of plasma carotenoids and recurrence risk (Supplementary Fig. 2). For fruit and vegetables combined, median intake was 247 (IQR 158–342) g/day prediagnosis, and 210 (IQR 135–299) at 3 months, and 216 (IQR 134–313) g/day at 15 months postdiagnosis. Median dietary carotenoid intake was 6288 (IQR 4127, 8936) and 6292 (IQR 4257–8837) µg/day, and median plasma carotenoid concentrations were 1353 (IQR 964-1772) nmol/L and 1355 (IQR 978–1847) nmol/L at 3 and 15 months postdiagnosis, respectively. The median age at diagnosis was 68 years, 80% were male, and 58% were diagnosed with high-risk NMIBC. Participants with higher compared to lower fruit and vegetable consumption at 3 months were more likely to be older, female, higher educated, never smoker, physically active, and to have a lower BMI (Table 1). Similar differences were observed for participants with higher compared to lower plasma carotenoid concentrations, but they were more likely to be younger (Supplementary Table 1). The median total follow-up time for the postdiagnosis analyses was 4.7 (IQR 3.3–5.2) years; 466 participants had one or more recurrences, 139 had two or more recurrences, and 153 had progression. 

**Table 1:** Descriptive characteristics of patients with NMIBC by quartiles of fruit and vegetable intake at 3 months postdiagnosis ^a^

Characteristic	Total	<135 g/day	135–210 g/day	210–300 g/day	≥ 300 g/day
N	1270	317	318	318	317
Fruit and vegetables (g/day)	210 (135, 299)	93 (60, 113)	171 (153, 190)	253 (231, 272)	375 (336, 441)
Vegetables (g/day)	98 (58, 144)	49 (23, 73)	92 (59, 126)	120 (81, 155)	159 (111, 205)
Fruits (g/day)	101 (45, 177)	26 (7, 56)	76 (46, 112)	130 (91, 173)	221 (177, 266)
Dietary total carotenoids (µg/day)	6288 (4127, 8936)	3795 (2498, 5380)	5886 (4214, 7709)	7386 (5312, 9928)	9127 (6799, 12,303)
Plasma total carotenoids (nmol/L) ^b^	1353 (964, 1772)	1162 (869, 1503)	1384 (1000, 1791)	1409 (983, 1784)	1544 (1130, 2087)
Age at diagnosis (years)	68 (62, 73)	67 (61, 72)	67 (61, 72)	68 (62, 73)	69 (64, 74)
Male	1022 (80%)	275 (87%)	249 (78%)	251 (79%)	247 (78%)
Education level ^c, d^					
Low	572 (46%)	151 (48%)	161 (52%)	136 (43%)	124 (39%)
Intermediate	342 (27%)	85 (27%)	83 (27%)	90 (29%)	84 (27%)
High	343 (27%)	80 (25%)	68 (22%)	88 (28%)	107 (34%)
Employment ^c, e^					
Employed	373 (30%)	94 (30%)	100 (32%)	108 (34%)	71 (23%)
Unemployed	24 (1.9%)	10 (3.2%)	6 (1.9%)	2 (0.6%)	6 (1.9%)
(Early) retirement	760 (60%)	187 (59%)	185 (59%)	183 (58%)	205 (65%)
Occupationally disabled	63 (5.0%)	17 (5.4%)	12 (3.8%)	12 (3.8%)	22 (7.0%)
Different	37 (2.9%)	8 (2.5%)	9 (2.9%)	9 (2.9%)	11 (3.5%)
Tumour stage					
Ta	697 (55%)	181 (57%)	187 (59%)	172 (54%)	157 (50%)
T1	548 (43%)	129 (41%)	125 (39%)	142 (45%)	152 (48%)
Tis	25 (2.0%)	7 (2.2%)	6 (1.9%)	4 (1.3%)	8 (2.5%)
Tumour grade ^f^					
1	210 (17%)	55 (17%)	48 (15%)	54 (17%)	53 (17%)
2	549 (43%)	137 (43%)	143 (45%)	138 (44%)	131 (41%)
3	508 (40%)	124 (39%)	126 (40%)	125 (39%)	133 (42%)
CIS, n (%)	180 (14%)	38 (12%)	49 (15%)	48 (15%)	45 (14%)
EAU risk score					
Low	225 (18%)	55 (17%)	59 (19%)	53 (17%)	58 (18%)
Intermediate	314 (25%)	89 (28%)	86 (27%)	79 (25%)	60 (19%)
High	731 (58%)	173 (55%)	173 (54%)	186 (58%)	199 (63%)
Initial treatment					
TURBT only	198 (16%)	48 (15%)	47 (15%)	56 (18%)	47 (15%)
TURBT + single instillation	304 (24%)	81 (26%)	85 (27%)	72 (23%)	66 (21%)
Chemotherapy	254 (20%)	61 (19%)	56 (18%)	72 (23%)	65 (21%)
BCG	514 (40%)	127 (40%)	130 (41%)	118 (37%)	139 (44%)
Comorbidity					
0	348 (27%)	89 (28%)	84 (26%)	85 (27%)	90 (28%)
1	383 (30%)	95 (30%)	106 (33%)	94 (30%)	88 (28%)
> = 2	539 (42%)	133 (42%)	128 (40%)	139 (44%)	139 (44%)
BMI (kg/m^2^) ^g^	26.3 (24.3, 29.0)	26.6 (24.3, 28.7)	26.4 (24.4, 29.1)	26.6 (24.5, 29.7)	25.7 (23.9, 28.4)
Smoking status					
Never	223 (18%)	39 (12%)	55 (17%)	57 (18%)	72 (23%)
Former	859 (68%)	211 (67%)	213 (67%)	223 (70%)	212 (67%)
Current	188 (15%)	67 (21%)	50 (16%)	38 (12%)	33 (10%)
Alcohol (g/day)	8 (1, 21)	9 (1, 24)	9 (1, 20)	8 (0, 19)	8 (1, 18)
Moderate-to-vigorous physical activity (min/week) ^c^	450 (180, 875)	420 (165, 810)	450 (150, 885)	420 (210, 815)	490 (210, 960)
Energy intake (kJ/day)	8590 (7,027, 10,345)	7758 (6229, 9826)	8358 (6930, 9971)	8741 (7313, 10,468)	9081 (7807, 11,018)

^a^ Median (IQR) or n (%); ^b^ Plasma carotenoids of 409 participants were not measured; ^c^ Data of 12–13 participants were missing/unknown; ^d^ Education level is divided into low (primary, secondary, and vocational education), medium (intermediate vocational, higher general secondary, and preuniversity education), and high (university of vocational education, university); ^e^ Different means no paid job due to other reasons such as scholar/student or full-time care of household; ^f^ Data of 3 participants were missing/unknown; ^g^ Data of 22 participants were missing/unknown

### Pre- and postdiagnosis fruit and vegetable consumption

**Fig. 1:**
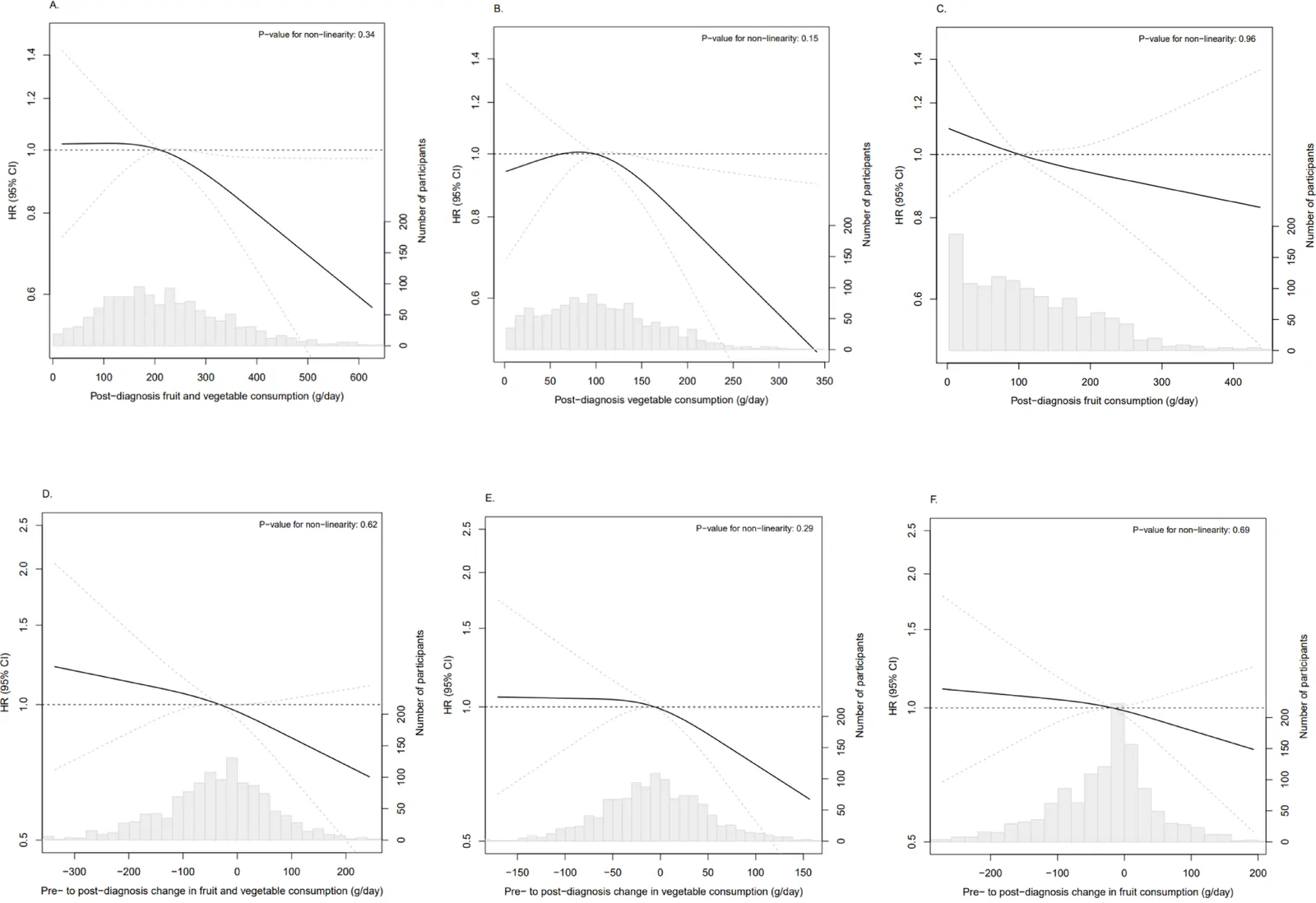
Restricted cubic splines for the association of fruit and vegetable consumption at 3 months postdiagnosis (**A**), vegetable consumption at 3 months postdiagnosis (**B**), fruit consumption at 3 months postdiagnosis (**C**), pre- to 3-month postdiagnosis change in fruit and vegetable consumption (**D**), pre- to 3-month postdiagnosis change in vegetable consumption (**E**), and pre- to 3-month postdiagnosis change in fruit consumption (**F**) with risk of first recurrence, with three knots at the 10th, 50th and 90th percentile of intake levels, and graphs truncated at the 1st and 99th percentile. HRs and corresponding 95%CI were estimated using multivariable Cox proportional hazards regression with adjustment for age at diagnosis, sex, cigarette smoking status (3 mo), BMI (3 mo), moderate-to-vigorous physical activity (3 mo), energy intake (3 mo or 15 mo), EAU risk score. The solid and dashed lines depict the HR and 95%CIs, respectively. The dashed line at HR = 1 depicts a null association. The distribution of intakes and changes is shown at the bottom

Higher prediagnosis total fruit and vegetable consumption, as well as vegetable consumption and fruit consumption separately, were not associated with risk of first recurrence and progression (Table 2). For 3 months after diagnosis, inverse associations with first recurrence risk were found for each 100 g/day increase in total fruit and vegetable (HR = 0.91, 95%CI 0.85–0.99) and vegetable intake (HR = 0.87, 95%CI 0.76–1.00) (Table 2). Splines showed that the linear associations were only significant for intakes above the median (Fig. 1). Associations for fruit consumption were in the same direction but not significant (HR = 0.92, 95%CI 0.84–1.02). The ICC between fruit and vegetable consumption at 3 and 15 months after diagnosis was 0.60 (Supplementary Table 2). At 15 months, only vegetable consumption tended to be inversely associated with first recurrence risk (HR = 0.88, 95%CI 0.76–1.02). For multiple recurrences, associations were attenuated and not statistically significant. No associations with progression were observed, except for an increased risk for higher fruit consumption at 15 months after diagnosis (HR = 1.15, 95%CI 1.03–1.29).

**Table 2:** Multivariable adjusted HRs and 95% CIs for the association of pre- and post-diagnosis fruit and vegetable intake^a^, dietary and plasma carotenoids^b^ with risks of first recurrence, multiple recurrences, and progression

Characteristic	First recurrence			Multiple recurrences			Progression		
	*N*	Events/ Person-years	HR (95% CI)	*N*	Events/ Person-years	HR (95% CI)	*N*	Events/ Person-years	HR (95% CI)
Pre-diagnosis intake^c^									
Fruit and vegetables	1396	521/4624	0.97 (0.91–1.04)	1396	761/5809	1.01 (0.96–1.07)	1432	170/5995	1.03 (0.92–1.16)^e^
Vegetables	1396	521/4624	0.95 (0.82–1.10)	1396	761/5809	0.98 (0.87–1.10)	1432	170/5995	0.97 (0.76–1.25)
Fruit	1396	521/4624	0.98 (0.90–1.06)	1396	761/5809	1.02 (0.96–1.10)	1432	170/5995	1.05 (0.91–1.21)
Post-diagnosis intake or concentrations at 3 mo^d^									
Fruit and vegetables	1270	466/4144	**0.91 (0.85–0.99)**	1270	686/5200	0.96 (0.90–1.02)	1306	153/5338	1.02 (0.90–1.15)^e^
Vegetables	1270	466/4144	0.87 (0.76-1.00)	1270	686/5200	0.91 (0.81–1.02)	1306	153/5338	0.95 (0.75–1.20)
Fruit	1270	466/4144	0.92 (0.84–1.02)	1270	686/5200	0.98 (0.91–1.06)	1306	153/5338	1.05 (0.90–1.22)
Dietary totalcarotenoids	1270	466/4144	1.00 (0.91–1.11)	1270	686/5200	1.00 (0.92–1.09)	1306	153/5338	1.04 (0.87–1.25)
Plasma totalcarotenoids	910	331/3101	1.00 (0.84–1.19)	910	500/3931	1.06 (0.92–1.21)	929	75/4059	1.03 (0.72–1.46)^e^
Post-diagnosis intake or concentrations at 15 mo^d^									
Fruit and vegetables	1270	466/4144	1.00 (0.92–1.07)^e^	1270	686/5200	1.01 (0.96–1.07)	1306	153/5338	1.11 (0.99–1.23)
Vegetables	1270	466/4144	0.88 (0.76–1.02)	1270	686/5200	0.92 (0.81–1.04)	1306	153/5338	0.97 (0.75–1.26)
Fruit	1270	466/4144	1.04 (0.96–1.14)^e^	1270	686/5200	1.05 (0.98–1.12)	1306	153/5338	**1.15 (1.03–1.29)**
Dietary totalcarotenoids	1270	466/4144	0.97 (0.87–1.08)	1270	686/5200	0.99 (0.91–1.08)	1306	153/5338	1.18 (0.97–1.44)
Plasma totalcarotenoids	910	331/3101	1.00 (0.85–1.19)	910	500/3931	1.03 (0.90–1.18)	929	75/4059	1.16 (0.79–1.71)
Pre- to 3 mo postdiagnosis 100 g/day increase in intake^d^									
Fruit and vegetables	1270	466/4144	**0.90 (0.81–0.99)**	1270	686/5200	0.93 (0.86-1.00)	1306	153/5338	0.99 (0.84–1.16)
Vegetables	1270	466/4144	0.86 (0.73–1.02)	1270	686/5200	0.95 (0.86–1.05)	1306	153/5338	0.96 (0.72–1.26)
Fruit	1270	466/4144	0.91 (0.81–1.03)	1270	686/5200	0.89 (0.78–1.03)	1306	153/5338	1.01 (0.83–1.22)
Pre- to 15 mo postdiagnosis 100 g/day increase in intake^d^									
Fruit and vegetables	1270	466/4144	1.02 (0.93–1.11)	1270	686/5200	0.88 (0.77–1.01)	1306	153/5338	1.10 (0.98–1.24)
Vegetables	1270	466/4144	0.87 (0.73–1.04)	1270	686/5200	1.00 (0.93–1.07)	1306	153/5338	0.98 (0.71–1.35)
Fruit	1270	466/4144	1.07 (0.97–1.19)	1270	686/5200	1.04 (0.96–1.12)	1306	153/5338	**1.14 (1.01–1.28)**

Estimates in bold were statistically significant with *P* ≤ 0.05

^a^ Estimates for risks are per 100 g/day increase in fruit and vegetables, vegetables, or fruit intake

^b^ Estimates for risks are per doubling in dietary or plasma carotenoids

^c^ Adjusted for age at diagnosis, sex, cigarette smoking status (6 wk), BMI (6 wk), moderate-to-vigorous physical activity (6 wk), energy intake (6 wk), EAU risk score

^d^ Adjusted for age at diagnosis, sex, cigarette smoking status (3 mo), BMI (3 mo), moderate-to-vigorous physical activity (3 mo), energy intake (3 mo or 15 mo), EAU risk score

^e^ Associations were non-linear, see Supplementary Fig. 4

### Postdiagnosis dietary and plasma carotenoids

The Pearson correlations between postdiagnosis fruit and vegetable consumption and dietary and plasma total carotenoids were 0.55 (data not shown) and 0.28 (Supplementary Table 3) at 3 months, and 0.55 and 0.24 at 15 months, respectively. The ICCs for total dietary and plasma carotenoids at 3 and 15 months after diagnosis were 0.53 and 0.79, respectively (Supplementary Table 2). Higher concentrations of total dietary and plasma carotenoids at 3 and 15 months after diagnosis were neither associated with risk of first recurrence, multiple recurrences, nor progression, with risk estimates around 1 (Table 2). For the 6 individual carotenoids, also no significant associations were found (Supplementary Tables 4 and 5).

### Stratified analyses

Significant associations for fruit and vegetable consumption at 3 months after diagnosis with first recurrence risk were only observed for women (HR = 0.80, 95%CI 0.66–0.95), former smokers (HR = 0.88, 95%CI 0.80–0.96), Ta tumour stage (HR = 0.84, 95%CI 0.85–0.99), low (HR = 0.81, 95%CI 0.67–0.99) or intermediate (HR = 0.84, 95%CI 0.72–0.99) EAU risk score, and no BCG treatment (HR = 0.87, 95%CI 0.79–0.95) (Table 3). Results were in the same direction but attenuated for data reported at 15 months.

For multiple recurrences, results were in the same direction as for first recurrence but attenuated (Supplementary Table 6). For progression, no clear differences by sex, smoking status, tumour stage, EAU risk score, and BCG treatment were observed for data reported at 3 months after diagnosis (Supplementary Table 7). For 15 months after diagnosis, increased risks for fruit consumption were only found for men (HR = 1.15, 95%CI 1.03–1.30), T1/Tis tumour stage (HR = 1.40, 95%CI 1.13–1.73) and high EAU risk score (HR = 1.15, 95%CI 1.02–1.30).

Estimates for dietary and plasma carotenoids in relation to recurrence were stronger for women than for men (diet: HR = 0.82, 95%CI 0.64–1.06 vs. HR = 1.04, 95%CI 0.93–1.16 and plasma: HR = 0.75, 95%CI 0.50–1.12 vs. HR = 1.06, 95%CI 0.87–1.28, respectively) but not statistically significant (Supplementary Table 8). No clear differences by smoking status, tumour stage, EAU risk score, and BCG treatment were observed. Findings for multiple recurrences and progression risk were similar (Supplementary Tables 9 and 10).

**Table 3:** Multivariable adjusted HRs and 95% CIs for the association of post-diagnosis fruit and vegetable intake with risks of first recurrence, stratified by sex, smoking status, tumour stage, EAU risk score, and BCG treatment

Characteristic	*N*	Events/person-years	Fruit and vegetables	Vegetables	Fruits
			HR (95% CI)	HR (95% CI)	HR (95% CI)
Post-diagnosis intake at 3 mo (per 100 g/day increase)^a^					
Overall	1270	466/4144	**0.91 (0.85–0.99)**	0.87 (0.76-1.00)	0.92 (0.84–1.02)
Sex					
Men	1022	367/3327	0.94 (0.86–1.03)	0.90 (0.77–1.06)	0.95 (0.86–1.06)
Women	248	99/818	**0.80 (0.66–0.95)**	**0.72 (0.52–0.99)**	0.82 (0.65–1.04)
Smoking status					
Never	223	81/753	1.08 (0.90–1.31)	1.02 (0.72–1.44)	1.12 (0.89–1.41)
Former	859	320/2769	**0.88 (0.80–0.96)**	**0.83 (0.70–0.98)**	0.89 (0.79-1.00)
Current	188	65/623	0.86 (0.69–1.09)	0.93 (0.64–1.35)	0.80 (0.58–1.10)
Tumour stage					
Ta	697	258/2340	**0.84 (0.75–0.93)**	**0.76 (0.62–0.92)**	**0.85 (0.74–0.98)**
T1/Tis	573	208/1815	1.00 (0.89–1.11)	1.00 (0.82–1.21)	0.99 (0.86–1.14)
EAU risk score					
Low	225	79/748	**0.81 (0.67–0.99)**	**0.62 (0.43–0.91)**	0.88 (0.69–1.13)
Intermediate	314	128/1029	**0.84 (0.72–0.99)**	**0.73 (0.55–0.98)**	0.87 (0.70–1.09)
High	731	259/2368	0.97 (0.88–1.07)	1.02 (0.86–1.22)	0.94 (0.83–1.06)
BCG					
Yes	514	156/1754	0.98 (0.87–1.12)	0.98 (0.77–1.23)	0.99 (0.84–1.16)
No	756	310/2390	**0.87 (0.79–0.95)**	**0.82 (0.68–0.97)**	**0.88 (0.77–0.99)**
Post-diagnosis intake at 15 mo (per 100 g/day increase)^a^					
Overall	1270	466/4144	1.00 (0.92–1.07)	0.88 (0.76–1.02)	1.04 (0.96–1.14)
Sex					
Men	1022	367/3327	1.04 (0.96–1.13)	0.94 (0.80–1.12)	1.08 (0.98–1.19)
Women	248	99/818	0.86 (0.73–1.03)	**0.66 (0.48–0.92)**	0.95 (0.75–1.20)
Smoking status					
Never	223	81/753	1.19 (0.99–1.43)	1.01 (0.72–1.41)	**1.36 (1.07–1.72)**
Former	859	320/2769	0.97 (0.88–1.06)	0.86 (0.71–1.03)	1.01 (0.91–1.13)
Current	188	65/623	0.89 (0.69–1.15)	0.83 (0.53–1.28)	0.90 (0.64–1.27)
Tumour stage					
Ta	697	258/2340	0.92 (0.82–1.02)	**0.79 (0.64–0.98)**	0.96 (0.84–1.09)
T1/Tis	573	208/1805	1.10 (0.98–1.23)	0.96 (0.78–1.19)	**1.20 (1.03–1.39)**
EAU risk score					
Low	225	79/748	0.94 (0.78–1.14)	0.77 (0.53–1.14)	1.02 (0.80–1.29)
Intermediate	314	128/1029	0.86 (0.73–1.02)	**0.71 (0.51–0.97)**	0.92 (0.75–1.14)
High	731	259/2368	1.06 (0.97–1.15)	1.00 (0.83–1.20)	1.09 (0.98–1.21)
BCG					
Yes	514	156/1754	1.07 (0.94–1.22)	0.97 (0.75–1.24)	1.14 (0.97–1.35)
No	756	310/2390	0.96 (0.87–1.05)	**0.83 (0.70-1.00)**	1.00 (0.90–1.12)

Estimates in bold were statistically significant with P ≤ 0.05

^a^ Adjusted for age at diagnosis, sex, cigarette smoking status (3 mo), BMI (3 mo), moderate-to-vigorous physical activity (3 mo), energy intake (3 mo or 15 mo), EAU risk score

### Pre- to postdiagnosis changes in fruit and vegetable consumption

Each 100 g/day pre- to 3 month postdiagnosis increase in total fruit and vegetable intake (HR = 0.90, 95%CI 0.81–0.99) and vegetable intake (HR = 0.86, 95%CI 0.73–1.02) was inversely associated with first recurrence risk (Table 2; Fig. 1). Associations for vegetable consumption and fruit consumption, separately, were in the same direction but not significant (HR = 0.86, 95%CI 0.73–1.02 and HR = 0.92, 95%CI 0.84–1.02, respectively). For pre- to 15 month post-diagnosis changes, only for vegetables an inverse but non-significant association with first recurrence risk was found (HR = 0.87, 95%CI 0.713–1.04). For multiple recurrences, associations were similar but slightly attenuated. No associations with progression were observed, except for an increased risk for a pre- to post-diagnosis increase in fruit consumption at 15 months (HR = 1.14, 95%CI 1.01–1.28).

## Discussion

Higher postdiagnosis fruit and vegetable consumption, and pre-to-postdiagnosis increases in intake, were associated with a decreased risk of first recurrence in patients with NMIBC. Stratified analyses showed that associations were only significant for women, former smokers, patients with less aggressive tumours, and patients not treated with BCG. Postdiagnosis dietary and plasma carotenoids were not associated with first recurrence risk. For multiple recurrences and progression, and for prediagnosis fruit and vegetable consumption, no clear associations were found.

Our finding that higher 3 months postdiagnosis but not prediagnosis fruit and vegetable consumption is associated with decreased NMIBC recurrence risk is largely in line with results from three previous studies [10–12]. The first study is a UK prospective cohort of 728 patients using a non-validated FFQ which also found no association between prediagnosis fruit and vegetable consumption and the risk of recurrence [10]. In contrast, fruit and vegetable consumption at 1 year after diagnosis was associated with decreased recurrence risk among 389 patients although results were not statistically significant (HR for third vs. first tertile 0.65, 95%CI 0.42–1.01) [10]. The second study was conducted among 595 patients from the US and reported that a prediagnosis dietary pattern high in fruits and vegetables was not associated with risk of recurrence [11]. The third study is a US prospective cohort of 1143 patients focusing on cruciferous vegetables intake and biomarkers of isothiocyanates (ITC) exposure measured shortly after diagnosis in relation to recurrence and progression [12]. This study showed no associations for cruciferous vegetable intake but an inverse association of plasma ITC-albumin adducts with progression risk. Overall, these results suggest that only consumption of fruits and vegetables after and not before NMIBC diagnosis may be important in relation to recurrence risk. This might be explained by the recurrence mechanisms of NMIBC [27] of which some might be altered by lifestyle. For example, fruit and vegetable consumption after diagnosis, but not necessarily before diagnosis, could impact reimplantation of tumour cells after TURBT by their immune-stimulating effects, and/or impact field cancerization (early genetic changes in a bladder epithelium field) [19]. These effects may be strongest shortly after TURBT, which may suggest that fruit and vegetable consumption at 3 months postdiagnosis is the most relevant exposure and could explain why results for 15 months were attenuated. Another explanation may be that prediagnosis fruit and vegetable consumption may be more prone to recall errors. Patients were asked to report their prediagnosis intake for summer and winter during the previous year, instead of during the previous month, which may be more prone to misclassificantion and attenuation of the risk estimates.

We further showed that associations of postdiagnosis fruit and vegetable consumption with NMIBC recurrence were only significant for women, former smokers, patients with less aggressive tumours, and patients not treated with BCG. These findings are partly in line with previous studies on BC risk. A meta-analysis on fruit and vegetable consumption and BC risk did not show differences by sex and smoking status [28]. However, a pooled analysis of 13 cohort studies only found an association of fruit [29] and vegetable [30] consumption with BC risk for women but not for men. The authors hypothesized that differences by sex may be explained by differences in hormones, somatic gene mutations, urination habits, behavioural vegetable intake, or residual confounding [29, 30]. Regarding smoking status, the pooled analysis showed that vegetable consumption was only associated with a decreased BC risk among current smokers [30] while for fruit consumption, no differences by smoking status were observed [29]. This may be explained by an interaction between vegetables and smoking in relation to bladder cancer risk, with phytochemicals present in vegetables potentially inhibiting smoking-related carcinogenesis [30]. Although the association with recurrence for current smokers in our study was not significant, it was in the same direction. For lower-risk and higher-risk NMIBC, the pooled analysis showed no different estimates fruit consumption [29] while this has not been specifically investigated for vegetable consumption [30]. An explanation for our finding that higher fruit and vegetable consumption was associated with a decreased recurrence risk among less aggressive NMIBC tumours only is currently lacking. However, we speculate this may be due to its impact on tumour reimplantation or field cancerization [27], which may be more efficient for less aggressive NMIBC tumours where the microenvironment may be still more responsive to immune modulation and DNA repair.

We found no association of dietary and plasma carotenoids with recurrence and progression risk. This has not been previously investigated in patients with NMIBC. However, a meta-analysis did find that the highest vs. lowest categories of dietary (RR = 0.88, 95%CI 0.76–1.03) and circulating (RR = 0.36, 95%CI 0.12–1.07) carotenoids were associated with a reduced BC risk [9], although results were not significant and there was significant heterogeneity. The risk estimate for circulating carotenoids was mainly driven by the case-control study of Hung et al. (RR = 0.11, 95%CI 0.05–0.26), while the nested case-control studies of Nomura et al. (RR = 0.54, 95%CI 0.23–1.27) and Ros et al. (RR = 0.70, 95%CI 0.41–1.18) did not show significant associations. Our null findings may be explained by the weak correlations between fruit and vegetable intake, dietary and plasma carotenoids. These ranged from − 0.02 to 0.48 but were similar to correlations reported by other studies [31–34]. It may be that other vitamins or phytochemicals in fruit and vegetables, for example folate [35], are more important in NMIBC recurrence and progression risk than carotenoids. However, this needs further investigation and the association of dietary and plasma carotenoids with NMIBC recurrence remains currently unclear.

There are some limitations of our study. First, fruit and vegetable consumption and dietary carotenoid intake were self-reported, and are difficult to assess due to seasonal differences in intake. Therefore, they may be prone to potential non-differential misclassification, leading to attenuation of the associations. This may be partially circumvented by the use of plasma carotenoids as biomarkers for fruit and vegetable consumption. However, these may not sufficiently reflect fruit and vegetable consumption due to variability in cooking methods for the preparation of carotenoid-rich foods, and the variability in carotenoid digestion and absorption [36]. This is substantiated by the weak correlations between fruit and vegetable intake and plasma carotenoids in our and other studies [31–34]. Plasma carotenoids may also not necessarily reflect urinary concentrations of carotenoids or their metabolites. Carotenoids are extensively metabolized, and plasma and tissue concentrations are determined by many factors, for example age, sex, and alcohol consumption [37]. These metabolites were not measured in the current study but may be more important in relation to NMIBC outcomes than the carotenoids. Second, we did not adjust plasma carotenoid concentrations for plasma lipids as these data were not available in our study. This has been previously recommended since carotenoids are transported in blood as part of lipoprotein complexes [38]. However, in the study on plasma carotenoids and breast cancer recurrence and death, additional adjustment for cholesterol concentrations did not change the risk estimates [39]. Therefore, we do not expect this to have substantially influenced our results. Third, we cannot exclude that some of our findings, such as the unexpected increased progression risk with higher fruit consumption at 15 but not 3 months postdiagnosis, are due to chance, since we studied many exposure variables and three outcomes and the number of events for progression analyses was limited. Lastly, we cannot rule out that participants and eligible non-participants differed in lifestyle, including fruit and vegetable consumption, potentially limiting the generalizability of our findings, although no significant differences in age, sex, and tumour characteristics were found [19].

Our study had several strengths. It is the largest study on (changes in) pre- and postdiagnosis fruit and vegetable consumption, and the first study on postdiagnosis dietary and plasma carotenoids in relation to NMIBC recurrence and progression risk to date. Although we cannot exclude the possibility of residual confounding, the detailed lifestyle and clinical follow-up data collection enabled the adjustment of analyses for relevant lifestyle and clinical factors selected using a DAG, as well as the analysis of multiple recurrence risk. Collection and analysis of data on postdiagnosis fruit and vegetable consumption and dietary and plasma carotenoids at two time points after diagnosis increased the robustness of our findings. ICCs for intake of fruit and vegetables and dietary carotenoids at 3 and 15 months after diagnosis were moderate (range 0.50–0.67) and ICCs for plasma carotenoids were good (range 0.72–0.81), with the latter being similar to two other studies showing 1-year ICCs from 0.56 to 0.75 [40] and 2–3 year ICCs from 0.73 to 0.88 [41], respectively. This suggests that fruit and vegetables consumption, and dietary and plasma carotenoids were relatively stable after diagnosis.

In conclusion, we found that higher postdiagnosis, and pre-to-post-diagnosis increases in fruit and vegetable consumption were associated with a decreased first recurrence risk in NMIBC. This association was only found in women, former smokers, patients with less aggressive tumours, and patients not treated with BCG. No clear associations for multiple recurrences and progression, and for prediagnosis fruit and vegetable consumption, were found. We found no associations for postdiagnosis dietary and plasma carotenoids. This is the first study on pre-to-postdiagnosis changes in fruit and vegetable consumption and postdiagnosis dietary and plasma carotenoids to date, and studies on fruit and vegetable consumption in relation to NMIBC recurrence and progression are scarce. Therefore, confirmation of our findings and studies investigating other biomarkers of fruit and vegetable consumption and their (bioactive) metabolites are necessary before definitive conclusions can be drawn and dietary recommendations can be provided.

## Supplementary Information

Below is the link to the electronic supplementary material.

Supplementary Material 1

## Data Availability

The data that support the findings of this study are available from the corresponding author (email: alina.vrieling@radboudumc.nl) upon reasonable request. The data can only be used to answer research questions that relate to the UroLife study. Therefore, requests for data access will be checked against the conditions for sharing the data as described in the signed informed consent.

## References

[1] Bray F, Laversanne M, Sung H, Ferlay J, Siegel RL, Soerjomataram I et al (2024) Global cancer statistics 2022: GLOBOCAN estimates of incidence and mortality worldwide for 36 cancers in 185 countries. CA Cancer J Clin 74(3):229–263. 10.3322/caac.21834 38572751

[2] Babjuk M, Burger M, Capoun O, Cohen D, Comperat EM, Dominguez Escrig JL et al (2022) European association of urology guidelines on non-muscle-invasive bladder cancer (Ta, T1, and Carcinoma in Situ). Eur Urol 81(1):75–94. 10.1016/j.eururo.2021.08.010 34511303

[3] Cambier S, Sylvester RJ, Collette L, Gontero P, Brausi MA, van Andel G et al (2016) EORTC Nomograms and risk groups for predicting recurrence, progression, and disease-specific and overall survival in non–muscle-invasive stage Ta–T1 urothelial bladder cancer patients treated with 1–3 Years of maintenance bacillus Calmette-Guérin. Eur Urol 69(1):60–69. 10.1016/j.eururo.2015.06.045 26210894

[4] Diet N (2018) Physical activity and bladder cancer. World Cancer Research Fund/American Institute for Cancer Research

[5] Xenou D, Tzelves L, Terpos E, Stamatelopoulos K, Sergentanis TN, Psaltopoulou T (2022) Consumption of fruits, vegetables and bladder cancer risk: a systematic review and meta-analysis of prospective cohort studies. Nutr Cancer 74(6):2003–2016. 10.1080/01635581.2021.1985146 34726552

[6] Steinmetz KA, Potter JD (1991) Vegetables, fruit, and cancer. II. Mechanisms. Cancer Causes Control 2(6):427–442. 10.1007/bf00054304 1764568

[7] Medina-García M, Baeza-Morales A, Martínez-Peinado P, Pascual-García S, Pujalte-Satorre C, Martínez-Espinosa RM et al (2025) Carotenoids and their interaction with the immune system. Antioxid (Basel). 10.3390/antiox14091111 PMC12466690 41009015

[8] Burri BJ, Clifford AJ (2004) Carotenoid and retinoid metabolism: insights from isotope studies. Arch Biochem Biophys 430(1):110–119. 10.1016/j.abb.2004.04.028 15325918

[9] Wu S, Liu Y, Michalek JE, Mesa RA, Parma DL, Rodriguez R et al (2020) Carotenoid intake and circulating carotenoids are inversely associated with the risk of bladder cancer: a dose-response meta-analysis. Adv Nutr 11(3):630–643. 10.1093/advances/nmz120 PMC7231589 31800007

[10] Jochems SHJ, van Osch FHM, Reulen RC, van Hensbergen M, Nekeman D, Pirrie S et al (2018) Fruit and vegetable intake and the risk of recurrence in patients with non-muscle invasive bladder cancer: a prospective cohort study. Cancer Causes Control 29(6):573–579. 10.1007/s10552-018-1029-9 PMC5938309 29667104

[11] Westhoff E, Wu X, Kiemeney LA, Lerner SP, Ye Y, Huang M et al (2018) Dietary patterns and risk of recurrence and progression in non-muscle-invasive bladder cancer. Int J Cancer 142(9):1797–1804. 10.1002/ijc.31214 29235103

[12] Wang Z, Kwan ML, Haque R, Goniewicz M, Pratt R, Lee VS et al (2023) Associations of dietary isothiocyanate exposure from cruciferous vegetable consumption with recurrence and progression of non-muscle-invasive bladder cancer: findings from the Be-Well Study. Am J Clin Nutr 117(6):1110–1120. 10.1016/j.ajcnut.2023.04.006 PMC10447500 37044209

[13] de Goeij L, Westhoff E, Witjes JA, Aben KK, Kampman E, Kiemeney LA et al (2019) The UroLife study: protocol for a Dutch prospective cohort on lifestyle habits in relation to non-muscle-invasive bladder cancer prognosis and health-related quality of life. BMJ open 9(10):e030396 10.1136/bmjopen-2019-030396 PMC6797314 31619424

[14] de Vries JH, de Groot LC, van Staveren WA (2009) Dietary assessment in elderly people: experiences gained from studies in The Netherlands. Eur J Clin Nutr 63(Suppl 1):S69–74. 10.1038/ejcn.2008.68 19190649

[15] Siebelink E, Geelen A, de Vries JH (2011) Self-reported energy intake by FFQ compared with actual energy intake to maintain body weight in 516 adults. Br J Nutr 106(2):274–281. 10.1017/s0007114511000067 21338536

[16] Streppel MT, de Vries JH, Meijboom S, Beekman M, de Craen AJ, Slagboom PE et al (2013) Relative validity of the food frequency questionnaire used to assess dietary intake in the Leiden Longevity Study. Nutr J 12:75. 10.1186/1475-2891-12-75 PMC3680188 23758629

[17] RIVM/Netherlands Nutrition Center (2011) Dutch Food Composition (NEVO) Table 2011. Netherlands Nutrition Center, The Hague, The Netherlands

[18] Beeren I, Kiebach J, Hof JP, Buffart LM, Aben KKH, Witjes JA et al (2025) Physical activity and risks of recurrence and progression among patients with non-muscle invasive bladder cancer. Int J Cancer 157(10):2049–2060. 10.1002/ijc.70030 PMC12439070 40583504

[19] van Zutphen M, Hof JP, Aben KK, Kampman E, Witjes JA, Alm Kiemeney L et al (2023) Adherence to lifestyle recommendations after non-muscle invasive bladder cancer diagnosis and risk of recurrence. Am J Clin Nutr 117(4):681–690. 10.1016/j.ajcnut.2022.12.022 36781128

[20] Babjuk M, Burger M, Compérat EM, Gontero P, Mostafid AH, Palou J et al (2019) European association of urology guidelines on non-muscle-invasive bladder cancer (TaT1 and Carcinoma In Situ)– 2019 update. Eur Urol 76(5):639–657. 10.1016/j.eururo.2019.08.016 31443960

[21] Harrell JFE (2015) Regression modeling strategies: with applications to linear models, logistic and ordinal regression, and survival analysis. Springer International Publishing : Imprint: Springer, Cham

[22] Westhoff E, Witjes JA, Fleshner NE, Lerner SP, Shariat SF, Steineck G et al (2018) Body mass index, diet-related factors, and bladder cancer prognosis: a systematic review and meta-analysis. Bladder Cancer 4(1):91–112. 10.3233/BLC-170147 PMC5798521 29430510

[23] Al-Delaimy WK, van Kappel AL, Ferrari P, Slimani N, Steghens JP, Bingham S et al (2004) Plasma levels of six carotenoids in nine European countries: report from the European prospective investigation into cancer and nutrition (EPIC). Public Health Nutr 7(6):713–722. 10.1079/phn2004598 15369608

[24] Gruber M, Chappell R, Millen A, LaRowe T, Moeller SM, Iannaccone A et al (2004) Correlates of serum lutein + zeaxanthin: findings from the third national health and nutrition examination survey. J Nutr 134(9):2387–2394. 10.1093/jn/134.9.2387 15333733

[25] Woodside JV, Young IS, Gilchrist SE, Vioque J, Chakravarthy U, de Jong PT et al (2013) Factors associated with serum/plasma concentrations of vitamins A, C, E and carotenoids in older people throughout Europe: the EUREYE study. Eur J Nutr 52(5):1493–1501. 10.1007/s00394-012-0456-8 23097178

[26] Allore T, Lemieux S, Vohl MC, Couture P, Lamarche B, Couillard C (2019) Correlates of the difference in plasma carotenoid concentrations between men and women. Br J Nutr 121(2):172–181. 10.1017/s0007114518003045 30392471

[27] Teoh JY, Kamat AM, Black PC, Grivas P, Shariat SF, Babjuk M (2022) Recurrence mechanisms of non-muscle-invasive bladder cancer - a clinical perspective. Nat Rev Urol 19(5):280–294. 10.1038/s41585-022-00578-1 35361927

[28] Vieira AR, Vingeliene S, Chan DS, Aune D, Abar L, Navarro Rosenblatt D et al (2015) Fruits, vegetables, and bladder cancer risk: a systematic review and meta-analysis. Cancer Med 4(1):136–146. 10.1002/cam4.327 PMC4312127 25461441

[29] Jochems SHJ, Reulen RC, van Osch FHM, Witlox WJA, Goossens ME, Brinkman M et al (2020) Fruit consumption and the risk of bladder cancer: a pooled analysis by the bladder cancer epidemiology and nutritional determinants study. Int J Cancer 147(8):2091–2100. 10.1002/ijc.33008 32285440

[30] Yu EY, Wesselius A, Mehrkanoon S, Goosens M, Brinkman M, van den Brandt P et al (2021) Vegetable intake and the risk of bladder cancer in the bladder cancer epidemiology and nutritional determinants (BLEND) international study. BMC Med 19(1):56. 10.1186/s12916-021-01931-8 PMC7942172 33685459

[31] Michaud DS, Giovannucci EL, Ascherio A, Rimm EB, Forman MR, Sampson L et al (1998) Associations of plasma carotenoid concentrations and dietary intake of specific carotenoids in samples of two prospective cohort studies using a new carotenoid database. Cancer Epidemiol Biomarkers Prev 7(4):283–290 9568782

[32] Jansen MC, Van Kappel AL, Ocké MC, Van ‘t Veer P, Boshuizen HC, Riboli E et al (2004) Plasma carotenoid levels in Dutch men and women, and the relation with vegetable and fruit consumption. Eur J Clin Nutr 58(10):1386–1395. 10.1038/sj.ejcn.1601981 15054421

[33] Al-Delaimy WK, Ferrari P, Slimani N, Pala V, Johansson I, Nilsson S et al (2005) Plasma carotenoids as biomarkers of intake of fruits and vegetables: individual-level correlations in the European Prospective Investigation into cancer and nutrition (EPIC). Eur J Clin Nutr 59(12):1387–1396. 10.1038/sj.ejcn.1602252 16160702

[34] Hendrickson SJ, Willett WC, Rosner BA, Eliassen AH (2013) Food predictors of plasma carotenoids. Nutrients 5(10):4051–4066. 10.3390/nu5104051 PMC3820058 24152746

[35] Tu H, Dinney CP, Ye Y, Grossman HB, Lerner SP, Wu X (2018) Is folic acid safe for non-muscle-invasive bladder cancer patients? An evidence-based cohort study. Am J Clin Nutr 107(2):208–216. 10.1093/ajcn/nqx019 PMC6669327 29529165

[36] Burrows TL, Williams R, Rollo M, Wood L, Garg ML, Jensen M et al (2015) Plasma carotenoid levels as biomarkers of dietary carotenoid consumption: a systematic review of the validation studies. J Nutr Intermed Metab 2(1):15–64. 10.1016/j.jnim.2015.05.001

[37] Moran NE, Mohn ES, Hason N, Erdman JW Jr., Johnson EJ (2018) Intrinsic and extrinsic factors impacting absorption, metabolism, and health effects of dietary carotenoids. Adv Nutr 9(4):465–492. 10.1093/advances/nmy025 PMC6054194 30032230

[38] Rock CL, Flatt SW, Natarajan L, Thomson CA, Bardwell WA, Newman VA et al (2005) Plasma carotenoids and recurrence-free survival in women with a history of breast cancer. J Clin Oncol 23(27):6631–6638 10.1200/JCO.2005.19.505 16170170

[39] Eliassen AH, Liao X, Rosner B, Tamimi RM, Tworoger SS, Hankinson SE (2015) Plasma carotenoids and risk of breast cancer over 20 y of follow-up. Am J Clin Nutr 101(6):1197–1205 10.3945/ajcn.114.105080 PMC4441811 25877493

[40] Al-Delaimy WK, Natarajan L, Sun X, Rock CL, Pierce JP (2008) Reliability of plasma carotenoid biomarkers and its relation to study power. Epidemiology 19(2):338 10.1097/EDE.0b013e3181635dc2 PMC2574518 18300693

[41] Kotsopoulos J, Tworoger SS, Campos H, Chung FL, Clevenger CV, Franke AA et al (2010) Reproducibility of plasma and urine biomarkers among premenopausal and postmenopausal women from the Nurses’ Health Studies. Cancer Epidemiol Biomarkers Prev 19(4):938–946. 10.1158/1055-9965.Epi-09-1318 PMC2852492 20332276

